# Genetic Evidence of Killer Whale Predation on White Sharks in Australia

**DOI:** 10.1002/ece3.70786

**Published:** 2025-01-27

**Authors:** Isabella M. Reeves, Andrew R. Weeks, Alison V. Towner, Rachael Impey, Jessica J. Fish, Zach S. R. Clark, Paul A. Butcher, Lauren Meyer, David M. Donnelly, Charlie Huveneers, Nicky Hudson, Adam D. Miller

**Affiliations:** ^1^ Cetacean Research Centre (CETREC WA) Perth Western Australia Australia; ^2^ Southern Shark Ecology Group, College of Science and Engineering Flinders University Adelaide South Australia Australia; ^3^ EnviroDNA Brunswick Victoria Australia; ^4^ School of BioSciences The University of Melbourne Parkville Victoria Australia; ^5^ Department of Ichthyology and Fisheries Science Rhodes University Makhanda South Africa; ^6^ South African International Maritime Institute Ocean Science Campus Gqeberha South Africa; ^7^ EcoGenetics Lab, School of Life and Environmental Sciences Deakin University Warrnambool Victoria Australia; ^8^ New South Wales Department of Primary Industries National Marine Science Centre Coffs Harbour New South Wales Australia; ^9^ Killer Whales Australia Mornington Victoria Australia; ^10^ Dolphin Research Institute Hastings Victoria Australia; ^11^ Gunditj Mirring Traditional Owners Aboriginal Corporation Heywood Victoria Australia

**Keywords:** Australia, DNA metabarcoding, selective consumption, top marine predator

## Abstract

Killer whales ( *Orcinus orca* ) have been documented to prey on white sharks ( *Carcharodon carcharias* ), in some cases causing localised shark displacement and triggering ecological cascades. Notably, a series of such predation events have been reported from South Africa over the last decade, with killer whales specifically targeting sharks' liver. However, observations of these interactions are rare, and knowledge of their frequency across the world's oceans remains limited. In October 2023, a 4.7 m (total length) white shark carcass washed ashore in southeastern Australia, coinciding with reports from citizen scientists of killer whales hunting a large, unidentified prey item in the area. Visual inspection of the carcass revealed that the liver, digestive, and reproductive organs were missing, and the presence of four distinctive bite wounds, one of which was characteristic of killer whale liver extraction as seen in South Africa. Genomic analyses performed on swabs taken from the bite wounds confirmed the presence of killer whale DNA in the major bite area, while the other bites were embedded with genetic material from the scavenging broadnose sevengill shark ( *Notorynchus cepedianus* ). These results provide confirmed evidence of killer whale predation on white sharks in Australia and the likely selective consumption of the liver, suggesting predations of this nature are more globally prevalent than currently assumed.

## Graphical abstract

Using a combination of wildlife forensics and citizen science, we provide the first evidence of killer whale predation on Australian white sharks, targeting the liver. This is the first time a predation event of this nature has been recorded outside of South Africa, suggesting they are perhaps more globally prevalent than currently assumed.

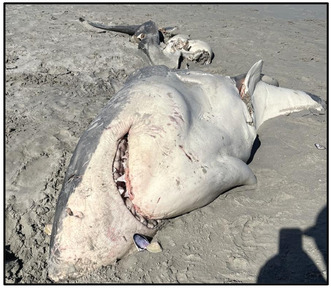

## 1 Introduction

Killer whales ( *Orcinus orca* ) are cosmopolitan predators with a broad distribution extending from the polar pack ice to the equator (Forney and Wade 2006). They are known to feed upon a diverse array of prey, including cetaceans, pinnipeds, cephalopods, teleosts, elasmobranchs, and reptiles (e.g., Baird et al. 2006; Pitman and Dutton 2004; Pitman et al. 2015; Remili et al. 2023; Visser 2005). Killer whales can exhibit geographical differences in foraging ecology, at times developing specialised diets and/or complex predatory behaviours (Ford et al. 1998; Pitman et al. 2015; Similä and Ugarte 1993). This includes selective consumption of specific prey tissues, such as whale tongue (Reeves et al. 2023; Scammon 1874) and shark livers (Ford et al. 2011).

Over the last decade, a series of predation events on white sharks (*Carcharodon carcharias*) by killer whales in South Africa, specifically targeting the liver, caused the abrupt displacement of white sharks in the region and subsequent ecosystem cascades (Bowlby et al. 2023; Towner et al. 2022, 2023). Killer whales have also been documented to prey on white sharks, at least occasionally, in California, resulting in similar shark flight responses and ecological cascades confirmed by quantifiable increases in white shark prey (Jorgensen et al. 2019; Pyle et al. 1999). However, direct observations of these predations are rare, and knowledge of their frequency remains limited.

In Australia, killer whales have been reported to prey on various shark species, such as blue sharks ( *Prionace glauca* ), porbeagles ( *Lamna nasus* ), shortfin makos ( *Isurus oxyrinchus* ), ground sharks (most likely school sharks, *Galeorhinus galeus* ), and tiger sharks ( *Galeocerdo cuvier* ), and to occasionally target the shark livers (Morrice 2004; Totterdell, 2016; Killer Whales Australia, unpublished data). Several interactions between killer whales and white sharks have been reported in Australia, including at least one suspected kill at the Neptune Islands Group Marine Park, South Australia, in February 2015 (www.youtube.com/watch?v=WC8Wxfn5xFw, Matt Waller, pers. comm.). On this occasion an oil slick indicative of a successful predation was observed following the interaction, although no carcass was recovered to confirm the kill.

In this study we use a combination of wildlife forensics and citizen science data to provide the first confirmed evidence of killer whale predation on white sharks in Australia. Genomic analyses were performed on biological samples taken from a beach‐cast white shark carcass from southeastern Australia, suspected to have succumbed to killer whale predation based on observations from citizen scientists. Results confirmed the presence of killer whale DNA in a bite wound characteristic of liver extraction by killer whale predation and genetic material from broadnose sevengill shark ( *Notorynchus cepedianus* ) in other bite wounds indicative of post‐kill scavenging. These findings are consistent with those previously reported from South Africa suggesting that predations of this nature are potentially more prevalent than currently assumed (Pyle et al. 1999; Towner et al. 2022).

## 2 Methods

### 2.1 Observation Description

**FIGURE 1:**
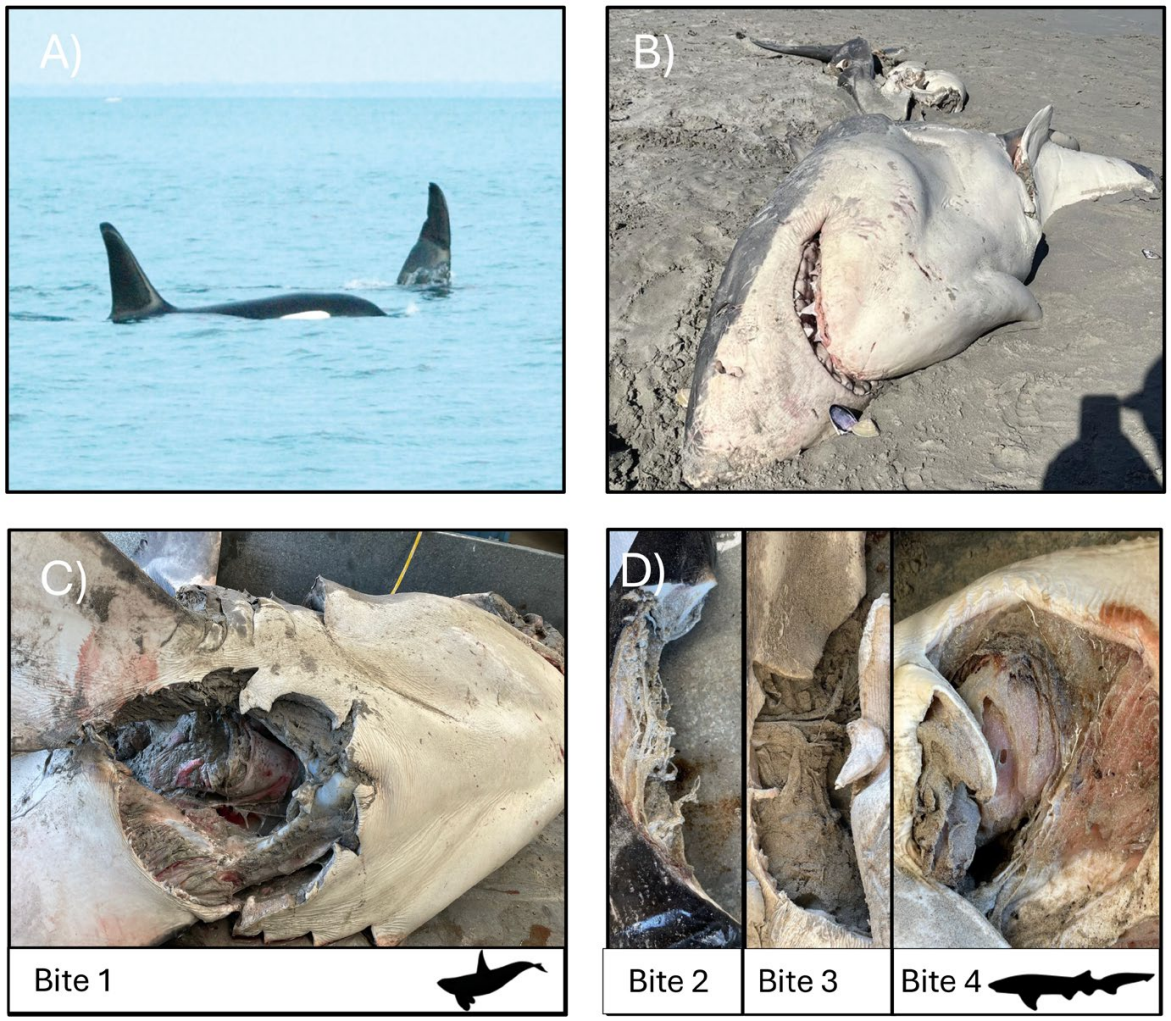
White shark carcass (*Carcharodon carcharias*) with bite wounds attributed to killer whale ( *Orcinus orca* ) predation. (A) Killer whales Ripple (EA_0005) and Bent Tip (EA_0007), known for their long‐term association, photographed together in Port Phillip Bay, Victoria, Australia, in 2015 (source: Karl Bromelow). (B) White shark carcass found at Bridgewater Bay, Victoria, Australia, on 17th October 2023 (source: Ben Johnson, Portland Bait and Tackle). (C) Bite wound at the pectoral girdle where killer whale DNA was detected (source: Adam Miller). (D) Bite wounds 2, 3, and 4 where broadnose sevengill shark DNA was found (source: Adam Miller).

On the 15th of October 2023, at least three killer whales were sighted at Bridgewater Bay, southeastern Australia, a region recognised as an important movement corridor for killer whales and white sharks (Bradford et al. 2020; Killer Whales Australia, unpublished data). Citizen scientists and experienced whale spotters reported confirmed sightings of catalogued killer whales Ripple (EA_0005) and Bent Tip (EA_0007), which have shown a long‐term association to each other (Figure 1A) and have been observed in the area on eight occasions between 2007 and 2023 (Killer Whales Australia, unpublished data). On this day both of these killer whales were also observed to be in association with another catalogued killer whale (EA_0037) (Killer Whales Australia, unpublished data). Anecdotal reports from the citizen scientists suggested the killer whales to be engaged in hunting behaviour, including the corralling and tossing of a large prey item (e.g., small whale, large shark) (Allen McCauley, pers. Comm., October 2023).

Subsequently, a large white shark washed ashore at Bridgewater Bay on the 17th of October 2023 (Figure 1B). The carcass sparked significant attention from the public due to its size, bite marks, and the speculation of it being the result of a killer whale predation. The white shark carcass was secured by state government fisheries officers on October 17th and transported to local facilities for subsequent investigation.

### 2.2 Observed Wounds on the Shark Carcass

Twenty‐four hours after the white shark carcass was secured, we determined the carcass to be 4.7 m total length, but the sex could not be determined due to the pelvic fin region and internal organs missing and presumably consumed. Despite the head, spine, fins (pectoral and dorsal), and tail being intact, the entire midsection of the animal was missing, including the liver, digestive, and reproductive organs (Figure 1B). Several bite wounds of different sizes were evident on the carcass, suggesting several potential marine predators. Most notably, a single large bite wound, approximately 500 mm in diameter, was evident in the pectoral girdle (Figure 1C). The large bite wound was consistent with those found on white shark carcasses in South Africa post killer whale predations, with a characteristic opening to the main body cavity on the underside of the shark and rake and/or stretch marks (see Towner et al. 2022). There were additional bite wounds on the carcass that appeared to be semi‐circular with serrated edges and occurred randomly over the carcass, indicative of some type of scavenging predator (Figure 1D).

### 2.3 Genetic Sampling and DNA Extraction

In an attempt to identify the predator(s), we collected genetic material from each bite wound by swabbing internal and external wound surfaces with a flocked cotton swab and preserving each in separate sterile 1.5 mL microcentrifuge tubes filled with ATL buffer (QIAGEN). We collected a total of 15 swabs from four distinct bite wounds, with each swab taken from a different area of each wound site. This included three swabs from the large bite characteristic of killer whale predation (bite wound 1; Figure 1C) and four swabs from the other three smaller bite wounds (bite wounds 2, 3, and 4; Figure 1D). Samples were subsequently transported to the laboratory on ice and stored at 4°C for 24 h prior to genetic analysis. Total genomic DNA was extracted from the swabs using a QIAGEN DNeasy Blood and Tissue Kit, following the manufacturer's protocol with minor modifications as specified by Clark et al. (2023). We performed four negative extraction controls in parallel (all extraction steps performed but without a swab sample) to control for potential cross‐sample contamination.

### 2.4 DNA Metabarcoding Library Preparation, Sequencing and Bioinformatic Analysis

Polymerase chain reaction (PCR) targeting the mitochondrial 12S rRNA gene (106 bp) was performed using a universal vertebrate PCR assay (Riaz et al. 2011; Table 1) following the laboratory workflow outlined by Clark et al. (2023). Briefly, PCR primer combinations were modified to include Illumina adapter tails at their 5′ ends (‘TCG GCA GCG TCA GAT GTG TAT AAG AGA CAG’ and ‘GTC TCG TGG GCT CGG AGA TGT GTA TAA GAG ACA G’ for forward and reverse primers, respectively) to enable the addition of Illumina dual‐index barcodes. Additionally, blocking primers were used to suppress human and white shark DNA amplification (Table 1). We performed all PCR reactions in duplicate for each sample with PCR reaction matrices and thermal cycling conditions being consistent with those described by Clark et al. (2023). A total of eight negative template PCR controls (no DNA extract added) and four negative DNA controls (extract from negative DNA extractions) were included in each step of the metabarcoding process through to sequencing to control for laboratory contamination.

**TABLE 1:** Details of the vertebrate 12 s primer pair (Riaz et al. 2011) and blocking primer pairs used to target vertebrate DNA from swab samples taken from the bite wounds on the white shark carcass.

Vertebrate marker	Sequence
Forward primer – 12S	5′ ACTGGGATTAGATACCCC 3′
Reverse primer – 12S	5′ TAGAACAGGCTCCTCTAG 3′
Human blocking primer – 12S	5′ ACCCCACTATGCTTAGCCCTAAACC 3′
White shark blocking primer – 12S	5′ ACCCTACTATGTCTAACCACAAACT 3′
Forward primer – 18S	5′ GTACACACCGCCCGTC 3′
Reverse primer – 18S	5′ TGATCCTTCTGCAGGTTCACCTAC 3′

Index PCR reaction matrices and thermal cycling conditions were again performed following Clark et al. (2023). Forward and reverse index primers provided dual indices in unique combinations, allowing demultiplexing of pooled products and Illumina sequencing adapters. PCR products were purified using 0.8× volume of AmpureBead XP (Beckman Coulter) and quantified using a Qubit dsDNA BR Assay Kit (Invitrogen). Indexed PCR products were subsequently normalised and pooled to create a single pooled library for sequencing. The resulting library was finally denatured and sequenced on the Illumina iSeq platform using the iSeq Version 2 kit (300 bp paired end), allowing for an average read depth of 5 × 10^4^ DNA sequence reads per sample.

We performed bioinformatic analyses of the resulting DNA sequences from each bite wound following the analytical pipeline of Clark et al. (2023). In summary, this involved the alignment of filtered DNA sequences against a reference library consisting of 12S rRNA haplotype sequences representing more than 25,000 vertebrate species, including reference haplotype sequences for all marine mammal and elasmobranch species known to be present in southeastern Australian waters found on GenBank (www.ncbi.nlm.nih.gov/genbank/).

### 2.5 Shotgun DNA Library Preparation, Sequencing and Bioinformatic Analysis

Australian killer whales comprise at least two distinct populations associated with northwestern and southwestern Australia (Reeves et al. 2022). Population assignment of the killer whale individual(s) could not be achieved using mitochondrial 12S haplotype sequences due to a lack of intraspecific variation at the locus. Consequently, we completed shotgun sequencing on DNA extractions from swabs that tested positive for killer whale DNA (based on the findings from DNA metabarcoding) to obtain low‐coverage sequence data for the nuclear and/or mitogenome to assist with individual discrimination and population assignment. Genomic libraries were constructed from the DNA extracts by Novogene (Singapore) using a NEBNext Ultra II DNA library prep kit and sequenced them on an Illumina NovaSeq X Plus (150 bp PE). Resulting DNA sequence reads were subsequently processed using the pipeline established by Reeves et al. (2023). Briefly, we applied a competitive mapping strategy by aligning filtered reads longer than 30 bp using BWA‐MEM v2.2.1 (Li 2013) to the Norwegian killer whale (mOrcOrc1.1; GCA_937001465.1; Foote et al. 2022) and white shark (GCF_017639515.1; Vertebrate Genome Project) reference genomes to discriminate between predator and host shotgun sequence data. We extracted reads that mapped to the killer whale genome, collapsed clonal reads using SAMtools v1.13 (Li et al. 2009), and masked repeat regions using BEDtools v2.26 (Quinlan 2014) based on regions identified by RepeatMasker v4.1 (Smit, Hubley, and Green 2004) and the Cetartiodactyl repeat library (Kohany et al. 2006). We then blasted the remaining regions against the NCBI database using Blastn v 2.7.1 (Chen et al. 2015) to confirm the probability of remaining sequences being representative of killer whale DNA and in an attempt to determine the population origin of the killer whale(s) by comparing sequences with reference sequences from killer whales found on Genbank (www.ncbi.nlm.nih.gov/genbank/).

## 3 Results and Discussion

Genomic analyses based on DNA metabarcoding revealed the presence of killer whale DNA in only the large pectoral girdle bite wound characteristic of liver extraction (bite wound 1; Figure 1C). Specifically, DNA recovered from two of the three swabs taken from bite wound 1 had a 100% match to killer whale reference sequences on GenBank (e.g., GenBank accession numbers GU187210.1 and KF418372.1). Genomic analyses also confirmed the presence of broadnose seven‐gill shark DNA in swabs from the other three smaller bite wounds (bite wounds 2, 3, and 4; Figure 1D), again having a 100% match to a species reference sequence on Genbank (e.g., Genbank accession number NC_022731.1). Host (white shark) DNA was detected in all swab samples, while the DNA of only two additional marine species was detected across all samples, including common teleost fishes in the region (snapper [ *Pagrus auratus* ] and herring cale [ *Olisthops cyanomelas* ]).

Multiple lines of evidence point to the presence of killer whale DNA being the result of a true predation event and not an artefact of contamination from the marine environment. True negative controls from the field were not available for analysis, but swabs taken from multiple bite wounds and multiple sites from each bite wound were effectively contrasted against each other to control for background levels of environmental DNA (eDNA) on the shark carcass. Additionally, multiple studies have demonstrated the rapid decay of eDNA in aquatic and terrestrial environments and short‐term detection limits (typically < 24 h), particularly when present in trace concentrations (Andruszkiewicz Allan et al. 2021; Ellis et al. 2022; Harrison, Sunday, and Rogers 2019). The potential for contamination is therefore unlikely, given the swab samples were collected from the carcass more than 24 h after its retrieval from the beach and the apparent lack of eDNA signals from other common and abundant marine species from this biodiverse region (Bennett et al. 2015). Instead, the detection of killer whale and broadnose sevengill shark DNA was likely due to high concentrations of cellular and/or extracellular genetic material. Finally, the fact that this genetic material was recovered from distinctive bite wounds being characteristic of the respective species and direct observations of a predation event involving killer whales and a large prey item further supports the notion that our signals are indeed the result of predation by akiller whale(s) and subsequent scavenging by a broadnose sevengill shark(s).

Unfortunately, efforts to confirm the population identity of the killer whale(s) through shotgun sequencing were futile. Shotgun sequencing produced a negligible number of killer whale reads (< 200), with a low mapping quality score across chromosomes (< 10) and no reads for the mitogenome. This is likely due to either low concentrations of killer whale DNA relative to white host DNA (which was found to dominate the sequencing outputs) or due to poor‐quality killer whale template DNA. Nonetheless, the killer whales identified from the sightings and likely predation event are catalogued individuals that have been recorded for almost two decades in southeastern Australian waters and are therefore representative of the population from this region.

Our findings add to the observations from the Neptune Islands in 2015 by providing the confirmation of killer whale predation on white sharks and likely targeting of their liver within Australian waters. This suggests that predation events of this nature are more prevalent than previously presumed, now being confirmed in South Africa, California, and Australia (Bowlby et al. 2023; Jorgensen et al. 2019; Pyle et al. 1999; Towner et al. 2022, 2023). Additionally, the identification of three bites by broadnose sevengill sharks provides genomic evidence of scavenging facilitated by killer whale's tissue selectivity. These findings complement other studies that have demonstrated the value of wildlife forensic approaches for understanding the behaviour of marine predators (see Kraft et al. 2021; Martin et al. 2024; van Bleijswijk et al. 2014) and add to a growing body of literature that is providing novel insights into killer whale interactions with elasmobranchs across the world's oceans.

## Author Contributions

**Isabella M. Reeves:** conceptualization (equal), formal analysis (equal), methodology (equal), project administration (equal), writing – original draft (lead), writing – review and editing (lead). **Andrew R. Weeks:** data curation (equal), formal analysis (equal). **Alison V. Towner:** conceptualization (equal), investigation (equal), writing – original draft (equal), writing – review and editing (equal). **Rachael Impey:** conceptualization (equal), data curation (equal), investigation (equal), methodology (equal), writing – original draft (equal), writing – review and editing (equal). **Jessica J. Fish:** conceptualization (equal), data curation (equal), investigation (equal), methodology (equal), writing – original draft (equal), writing – review and editing (equal). **Zach S. R. Clark:** conceptualization (equal), data curation (equal), formal analysis (equal), investigation (equal), methodology (equal), writing – original draft (equal), writing – review and editing (equal). **Paul A. Butcher:** conceptualization (equal), writing – original draft (equal), writing – review and editing (equal). **Lauren Meyer:** conceptualization (equal), writing – original draft (equal), writing – review and editing (equal). **David M. Donnelly:** writing – original draft (equal), writing – review and editing (equal). **Charlie Huveneers:** conceptualization (equal), investigation (equal), writing – original draft (equal), writing – review and editing (equal). **Nicky Hudson:** directions from First Nations on the significance of the event, and preservation of the carcass. **Adam D. Miller:** conceptualization (equal), data curation (equal), formal analysis (equal), funding acquisition (equal), investigation (equal), methodology (equal), project administration (lead), writing – original draft (lead), writing – review and editing (lead).

## Conflicts of Interest

The authors declare no conflicts of interest.

## Data Availability

Demultiplexed, unfiltered reads (fastq), ASV haplotype sequences, sample metadata, and bioinformatic code that supports the findings of this study have been uploaded to Dryad (https://doi.org/10.5061/dryad.r2280gbn5).
